# 伴肾损害的初诊多发性骨髓瘤患者肾功能疗效影响因素

**DOI:** 10.3760/cma.j.issn.0253-2727.2023.02.010

**Published:** 2023-02

**Authors:** 宇航 宋, 富婧 张, 蓉蓉 胡, 苗 陈, 辰 杨, 为 王, 岩 秦, 道斌 周, 俊玲 庄

**Affiliations:** 1 中国医学科学院、北京协和医学院北京协和医院血液内科 100730 Department of Hematology, Peking Union Medical College Hospital, Chinese Academy of Medical Science and Peking Union Medical College, Beijing 100730, China; 2 中国医学科学院、北京协和医学院北京协和医院肾内科 100730 Department of Nephrology, Peking Union Medical College Hospital, Chinese Academy of Medical Science and Peking Union Medical College, Beijing 100730, China

**Keywords:** 多发性骨髓瘤, 肾功能, 高钙血症, 血液学反应, 1q21扩增, Multiple myeloma, Renal function, Hypercalcemia, Hematological response, 1q21 amplification

## Abstract

**目的:**

探究初诊伴肾损害的多发性骨髓瘤（MM）患者肾功能疗效的影响因素。

**方法:**

纳入2007年8月至2021年10月北京协和医院伴肾损害的MM患者181例，其基线慢性肾脏病（CKD）分期为3～5期。对不同肾功能疗效组患者的实验室检查、治疗方案、血液学反应深度、生存情况等进行分析。多因素分析采用Logistic回归模型。

**结果:**

纳入181例初诊时即有肾功能损害患者，以277例肾功能正常（CKD分期1～2期）患者作为对照组。大多数患者应用BCD和VRD方案。与无肾损害患者相比，肾损害患者的无进展生存（PFS）时间（14.0个月对24.8个月，*P*<0.001）和总生存（OS）时间（49.2个月对79.7个月，*P*<0.001）均显著缩短。肾功能疗效有效组和无效组高钙血症（*P*＝0.013，*OR*＝5.654）、1q21+（*P*＝0.018，*OR*＝2.876）和血液学反应达部分缓解及以上（*P*＝0.001，*OR*＝4.999）的差异有统计学意义，且均为肾功能疗效的独立预后因素。治疗后肾功能有效者的PFS时间和OS时间均较治疗后肾功能无效者延长（PFS：15.6个月对10.2个月，*P*＝0.074；OS：56.5个月对47.3个月，*P*＝0.665），差异无统计学意义。

**结论:**

高钙血症、1q21+和血液学反应深度是MM患者肾功能改善的独立预后因素。基线CKD分期3～5期的MM患者生存更差，治疗后肾功能疗效有效有助于改善PFS。

## Abstract (en)

**Objective:**

To investigate the causative factors of renal function in newly diagnosed multiple myeloma（MM）patients with renal inadequacy.

**Methods:**

181 MM patients with renal impairment from August 2007 to October 2021 at Peking Union Medical College Hospital were recruited, whose baseline chronic kidney disease（CKD）stage was 3–5. Statistical analysis was performed based on laboratory tests, treatment regimens, hematological responses, and survival among various renal function efficacy groups. A logistic regression model was employed in multivariate analysis.

**Results:**

A total of 181 patients were recruited, and 277 patients with CKD stages 1–2 were chosen as controls. The majority choose the BCD and VRD regimens. The progression-free survival（PFS）（14.0 months *vs* 24.8 months, *P*<0.001）and overall survival（OS）（49.2 months *vs* 79.7 months, *P*<0.001）of patients with renal impairment was considerably shorter. Hypercalcemia（*P*＝0.013, *OR*＝5.654）, 1q21 amplification（*P*＝0.018, *OR*＝2.876）, and hematological response over a partial response（*P*＝0.001, *OR*＝4.999）were independent predictive factors for renal function response. After treatment, those with improvement in renal function had a longer PFS than those without（15.6 months *vs* 10.2 months, *P*＝0.074）, but there was no disparity in OS（56.5 months *vs* 47.3 months, *P*＝0.665）.

**Conclusion:**

Hypercalcemia, 1q21 amplification, and hematologic response were independent predictors of the response of renal function in NDMM patients with renal impairment. MM patients with CKD 3–5 at baseline still have worse survival. Improvement in renal function after treatment is attributed to the improvement in PFS.

肾损害是多发性骨髓瘤（MM）最常见的并发症和临床表现之一，25％～50％的MM患者在病程中伴有不同程度的肾损害[1]。尽管新药治疗已经极大地改善了MM患者的疗效和总生存（OS），但肾功能不全患者的OS率显著低于肾功能正常的患者[2]，治疗后肾功能恢复患者的生存显著优于肾功能未恢复患者[3]。因此，即使在新药时代，治疗后肾功能无改善仍是生存的不利因素，说明肾脏的器官疗效具有预后意义。

骨髓瘤肾损害的机制复杂，包括管型肾病、高钙血症、继发轻链沉积病及淀粉样变性等，尽管有效化疗是改善肾脏功能的根本方法，但来那度胺等经肾脏代谢的化疗药物也可能加重肾脏损伤。由于影响因素众多，如何预测MM患者肾功能转归仍缺乏大样本数据。我们对北京协和医院数据库中伴肾损害的初诊MM（NDMM）患者的临床资料进行回顾性分析，拟探究NDMM患者肾功能疗效的影响因素，旨在为肾脏器官疗效提供预测依据。

## 病例与方法

1. 病例：回顾性纳入2007年8月至2021年10月于北京协和医院血液内科就诊的伴有肾损害的NDMM患者181例，诱导治疗期间至少记录两次估测肾小球滤过率（eGFR）。纳入同期基线肾功能正常［慢性肾脏病（CKD）分期1～2期］的NDMM患者作为生存分析的对照组。

2. 定义和诊断标准：MM诊断标准参照国际骨髓瘤工作组（IMWG）2014年的指南[4]。骨髓瘤肾损害定义为首诊时eGFR<60 ml·min^−1^·（1.73 m^2^）^−1^，其中eGFR的计算采用国际骨髓瘤工作组（IMWG）推荐的MDRD公式[5]。肾功能疗效的定义基于2016年IMWG标准[5]，肾功能治疗有效包括肾功能完全缓解（CR）、部分缓解（PR）、微小缓解（MR）以及首诊eGFR在50～60 ml·min^−1^·（1.73 m^2^）^−1^而诱导治疗期间最佳eGFR在60 ml·min^−1^·（1.73 m^2^）^−1^以上的患者，其他定义为肾功能无效，其中CKD分期治疗前后不变定义为稳定，变差定义为恶化。

3. 临床特征和分组：收集患者年龄、性别、修订的国际预后分期（R-ISS）、M蛋白类型、首诊的eGFR、乳酸脱氢酶（LDH）、血清钙、细胞遗传学特征、一线治疗方案等基线信息，记录基线CKD分期和肾功能转归状况。收集患者随访中的eGFR和血液学反应深度结果，并根据基线eGFR和诱导治疗期间最佳eGFR进行分组。比较不同肾功能疗效组间的生存差异。

4. 随访和生存分析：中位随访时间为62.30（1.63～153.04）个月，末次随访截至2021年10月。OS时间定义为患者自确诊MM至因任何原因死亡或失访的时间。无进展生存（PFS）时间定义为患者确诊MM至首次血液学疗效为疾病进展或复发的时间。血液学疗效客观缓解率（ORR）定义为血液学疗效达到PR及以上［包括PR、非常好的PR（VGPR）、CR和严格意义的CR（sCR）］患者所占比例。所有疗效评估参照2016版IMWG标准[6]。

5. 统计学处理：本研究所有的统计学分析均使用SPSS软件（IBM，version 26.0）、R4.1.2软件及Graphpad Prism 8.0.1软件完成。计量资料用中位数（范围）表示，计数资料用例数（百分比）表示。分类变量的组间比较采用卡方检验或Fisher确切概率法。连续性变量的比较采用单因素方差分析（ANOVA）。采用Kaplan-Meier法绘制生存曲线，与PFS和OS相关的单因素分析采用Log-rank检验。多因素分析采用Logistic回归模型，Logistic回归模型的构建采用LR法通过拟合优度筛选变量。*P*<0.05为差异有统计学意义。

## 结果

1. 临床特征：共纳入181例NDMM患者，基线资料见表1。其中男108例，女73例，中位年龄63（31～89）岁，按照肾功能CKD分期分为CKD3、CKD4、CKD5组，分别有患者60、59、62例。CKD3、CKD4、CKD5组患者IgH易位比例（58.3％对62.2％对33.3％）的差异有统计学意义（*P*＝0.016）。三组患者中147例（81.2％）一线治疗方案采用含硼替佐米的方案（硼替佐米+来那度胺+地塞米松或硼替佐米+环磷酰胺+地塞米松）。而三组患者的年龄、性别、R-ISS分期、M蛋白类型、LDH升高比例、高钙血症比例、其他细胞遗传学特征（包括1q21+，13q−，RB1−，17p−）、合并淀粉样变比例等基线特征的差异无统计学意义。

**表1:** 伴有肾损害的初诊多发性骨髓瘤患者的基线临床特征［例（％）］

临床特征	总体（181例）	CKD3（60例）	CKD4（59例）	CKD5（62例）	*χ*^2^值/*F*值	*P*值
年龄［岁，*M*（范围）］	63（31～89）	65（33～89）	63（31～86）	61（39～83）	1.381	0.254
男性［例（％）］	108（59.7）	31（51.7）	40（67.8）	37（59.7）	2.802	0.249
R-ISS分期［例（％）］					2.255	0.689
Ⅰ	1（0.6）	1（1.9）	0（0）	0（0）		
Ⅱ	76（45.0）	24（45.3）	26（44.1）	26（44.1）		
Ⅲ	92（54.4）	28（52.8）	33（55.9）	31（55.9）		
M蛋白类型［例（％）］					14.921	0.061
IgG	73（40.3）	27（45.0）	26（44.0）	20（32.2）		
IgA	35（19.3）	15（25.0）	11（18.6）	9（14.5）		
轻链型	58（32.0）	13（21.7）	20（33.9）	25（40.3）		
IgD	12（6.6）	3（5.0）	1（1.7）	8（12.9）		
其他	3（1.7）	2（3.3）	1（1.7）	0（0）		
高钙血症^a^［例（％）］	37（22.7）	9（18.3）	12（25.4）	16（24.2）	2.755	0.252
LDH升高^b^［例（％）］	40（22.1）	10（16.7）	15（25.4）	15（24.1）	3.458	0.484
细胞遗传学［例（％）］						
1q21+	59（40.0）	17（35.4）	18（34.8）	24（50.0）	3.742	0.442
IgH易位	76（51.8）	28（58.3）	32（61.8）	16（33.3）	11.668	0.016
17p−	18（12.4）	6（12.8）	7（15.0）	5（11.1）	0.812	0.904
淀粉样变［例（％）］	20（11.0）	8（13.3）	5（8.5）	7（11.3）	0.723	0.907
治疗方案［例（％）］					19.286	0.001
BCD	124（68.5）	29（48.3）	43（72.9）	52（83.9）		
VRD	24（13.3）	13（21.7）	8（13.6）	3（4.8）		
其他	33（18.2）	18（30.0）	8（13.6）	7（11.3）		

**注** ^a^血钙>2.70 mmol/L；^b^LDH>250 U/L；BCD：硼替佐米+环磷酰胺+地塞米松；VRD：硼替佐米+来那度胺+地塞米松

2. 肾功能转归状况：CKD3、CKD4、CKD5组患者诱导治疗期间肾功能疗效好转的例数分别为29例（48.3％）、46例（77.9％）和38例（61.3％），稳定的例数分别为27例（45.0％）、7例（11.9％）和24例（38.7％），恶化的例数分别为4例（6.7％）、6例（10.2％）和0例（0），三组间的差异有统计学意义（*χ*^2^＝21.750，*P*＝0.001）。

3. 肾功能疗效的单因素分析：单因素分析表明，影响肾功能疗效的因素包括高钙血症（*P*＝0.003）、血液学反应深度达到PR及以上（*P*＝0.001）、深度缓解（*P*＝0.012），1q21+的差异无统计学意义（*P*＝0.058），但可能是影响肾功能疗效的潜在因素（表2）。

**表2:** 不同肾功能疗效组初诊多发性骨髓瘤患者的临床特征比较

临床特征	无效组（68例）	有效组（113例）	*χ*^2^值/*F*值	*P*值
男性［例（％）］	35（51.5）	72（63.7）	4.583	0.109
年龄［岁，*M*（范围）］	62.5（33～89）	63（31～83）	1.425	0.243
R-ISS分期［例（％）］			3.706	0.106
Ⅰ	0（0）	1（0.9）		
Ⅱ	34（54.0）	42（39.6）		
Ⅲ	29（46.0）	63（59.4）		
M蛋白类型［例（％）］			5.471	0.242
IgG	25（36.8）	48（42.5）		
IgA	10（14.7）	25（22.1）		
轻链	27（39.7）	31（27.4）		
IgD	4（5.9）	8（7.1）		
其他	2（2.9）	1（0.9）		
LDH升高^a^［例（％）］	14（20.6）	26（23.0）	1.779	0.411
游离轻链［mg/L，*M*（范围）］	7 887.5（6.9～47 550.0）	7 812.4（7.9～41 100.0）	0.555	0.458
游离轻链比值［*M*（范围）］	226.6（1.1～3 995.8）	668.4（1.1～17 451.0）	1.408	0.238
高钙血症^b^［例（％）］	6（8.8）	31（27.4）	9.041	0.003
细胞遗传学［例（％）］				
1q21+	16（32.0）	43（44.8）	5.698	0.058
IgH易位	24（48.0）	52（53.1）	5.283	0.071
17p−	7（12.1）	11（12.2）	0.431	0.965
淀粉样变［例（％）］	9（13.6）	11（10.1）	0.559	0.753
疗效达PR及以上［例（％）］	29（44.6）	80（71.4）	14.043	0.001
深度缓解^c^［例（％）］	19（27.9）	53（46.9）	6.372	0.012
治疗方案［例（％）］			1.105	0.576
BCD	44（64.7）	80（70.8）		
VRD	9（13.2）	15（13.3）		
其他	15（22.1）	18（15.9）		
自体造血干细胞移植［例（％）］	7（10.3）	13（11.5）	0.721	0.929
含硼替佐米方案疗程数［*M*（范围）］	6.5（1～26）	6（3～27）	0.704	0.941

**注** ^a^LDH>250 U/L；^b^血钙>2.70 mmol/L；^c^血液学疗效达到非常好的部分缓解及以上；PR：部分缓解；BCD：硼替佐米+环磷酰胺+地塞米松；VRD：硼替佐米+来那度胺+地塞米松

4. 肾功能疗效的多因素Logistic回归分析：将上述单因素分析中差异有统计学意义的因素（高钙血症、疗效达PR及以上）和临床中关心的其他因素（包括细胞遗传学因素、年龄、性别、M蛋白类型、R-ISS分期）纳入Logistic回归模型，利用LR法进行变量拟合优度筛选，结果表明高钙血症（*OR*＝5.654，95％*CI* 1.311～10.080，*P*＝0.013）、1q21+（*OR*＝2.876，95％*CI* 1.377～6.007，*P*＝0.018）和疗效达PR及以上（*OR*＝4.999，95％*CI* 2.921～12.324，*P*＝0.001）为肾功能疗效的独立预测因素。

5. 生存分析：基线伴和不伴肾损害患者的生存分析表明，伴肾损害组和不伴肾损害组中位OS时间（49.2个月对79.7个月，*P*<0.001）、PFS时间（14.0个月对24.8个月，*P*<0.001）的差异均有统计学意义（图1）。

**图1:**
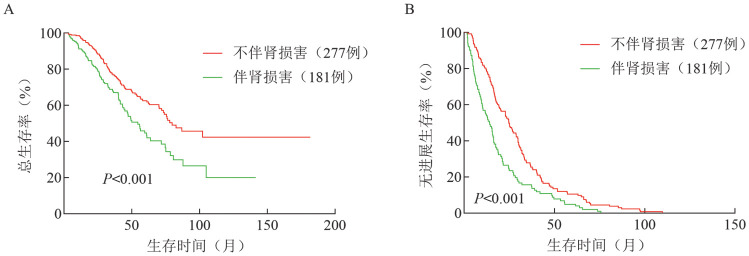
初诊伴肾损害和不伴肾损害组的总生存（A）和无进展生存（B）曲线

肾功能疗效无效和有效组中位OS时间（47.3个月对56.5个月，*P*＝0.665）、PFS时间（10.2个月对15.6个月，*P*＝0.074）的差异无统计学意义（图2）。

**图2:**
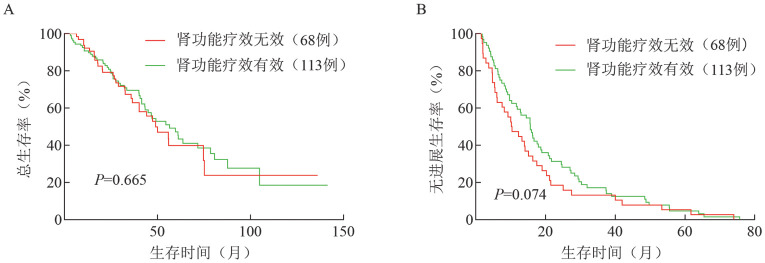
肾功能疗效无效和有效组的总生存（A）和无进展生存（B）曲线

## 讨论

肾损害是MM最常见的临床并发症之一，严重影响患者生活质量和OS，治疗后肾功能未恢复的患者预后更差。随着治疗MM的新药不断涌现，NDMM患者的疗效不断改善，生存期已延长至8～10年。但新药时代肾损害对患者生存的影响及器官功能改善的预测因素仍缺乏大样本数据，国内鲜有报道。因此，本研究拟探究NDMM患者肾功能疗效的预测因素，以评价肾脏疗效对预后的影响。本研究显示，181例伴肾损害的NDMM患者的PFS和OS均显著劣于基线不伴肾损害的患者。虽然治疗后肾功能是否改善的患者PFS差异无统计学意义（*P*＝0.074），但两组患者PFS的生存曲线已经明显分开，治疗后肾功能改善的患者PFS时间延长，且随着随访时间的延长可能显示出差异有统计学意义。本研究中单因素分析和多因素分析均显示，高钙血症、1q21+和疗效达PR及以上是肾功能改善的独立预后因素。

MM肾损害的机制复杂，主要可分为轻链介导的肾损害和非轻链介导的肾损害。轻链介导的肾损害包括管型肾病、继发淀粉样变、继发轻链沉积病等，非轻链介导的肾损害包括高钙血症、反复泌尿系统感染等。Katagiri等[7]在2011年报道，能否摆脱透析依赖患者的ISS分期、血液学反应深度、β_2_-微球蛋白、血清钙和肌酐水平差异有统计学意义。本研究将肾损害定义扩大至CKD分期3～5期，结果发现，伴有高钙血症的肾损害患者治疗后肾功能更易恢复（*OR*＝3.65，*P*＝0.013）。主要原因可能是MM治疗方案有效率高，迅速降低血钙，从而减轻了肾脏损害。CKD分期3～5期患者应用双膦酸盐受到影响，伴有高钙血症的MM患者联合应用地舒单抗可能对逆转肾功能损伤更有帮助[8]–[9]。

1q21+是骨髓瘤中最常见的与疾病进展和耐药相关的高危细胞遗传学因素[10]，在多项大型随机对照研究中被证实与不良预后相关[11]–[14]。存在1q21+的患者的M蛋白类型更倾向于IgA型，与更高的LDH、β_2_-微球蛋白水平相关[15]，更容易发生贫血、血小板减低、高钙血症等[16]。然而，1q21+和骨髓瘤肾损害的相关性研究鲜见报道。本研究发现，1q21+患者治疗后肾功能疗效有效的比例显著高于肾功能疗效无效的比例（*OR*＝2.876，*P*＝0.018），说明在肾脏疗效方面，1q21+并不是肾脏疗效不佳的危险因素。本中心前期研究也显示单一1q21+患者并非预后不良，只有合并其他高危遗传学异常时才导致OS时间缩短[17]。伴有1q21+的肾损害患者为何更易获得器官疗效改善仍需大样本数据进一步证实。

本研究队列中大多数患者采用BCD或VRD方案，肾功能有效和无效组中各种方案所占比例的差异无统计学意义。血液学反应深度达到PR及以上的患者肾功能疗效更佳（*OR*＝5.99，*P*<0.001），与文献报道相符[7]，提示有效的抗骨髓瘤治疗仍是改善肾损害最重要的手段。VRD方案是目前各指南推荐的最常用的一线治疗方案，诱导阶段ORR高达97％[18]–[19]，接受自体造血干细胞移植（ASCT）巩固和来那度胺维持治疗的患者首次PFS时间长达61个月。尽管来那度胺可以根据eGFR调整剂量[20]，国内很多医师对合并肾损害患者应用来那度胺仍有顾虑，肾损害患者行ASCT比例更低。从本中心数据也能看出，医师更多选用BCD方案，CKD分期5期患者VRD方案的应用比例仅为4.8％，这可能是肾损害患者生存期较CKD分期1～2期患者缩短的原因之一。一项纳入26例肾损害患者的药代动力学研究中，应用根据eGFR调整剂量的VRD方案后，ORR仍达到77％，其中包括CKD分期5期患者和已经发展为终末期肾脏病的患者，表明VRD方案仍可能在肾损害的患者中表现出良好的诱导治疗结局，但需要严格根据肾功能调整来那度胺的用量[21]。此外，单克隆抗体如达雷妥尤单抗治疗伴肾损害的MM患者无需调整剂量，即使在透析患者中也可应用[22]–[23]，一线应用较对照组显著改善肾损害患者的生存。

即使骨髓瘤治疗方案不断优化，肾损害仍然是骨髓瘤预后的重要影响因素，有肾损害患者的中位PFS时间较无肾损害患者显著降低（14.0个月对24.8个月）。另外，治疗后肾功能改善患者的中位PFS时间较肾功能无效的患者延长（15.6个月对10.2个月），表明可以用肾功能疗效预测患者的预后。

肾损害病程也可能是影响MM患者肾功能恢复的因素之一。本研究提示合并高钙血症的患者肾功能更易恢复，表明急性肾损伤（AKI）时肾功能恢复得更快。由于MM多发于老年人，合并心血管疾病的概率高，基础肾脏损害会进一步增加治疗的难度和复杂性。鲜有研究报道肾损害持续时间与肾功能恢复的相关性，有研究显示透析时间并不能预测MM患者治疗后能否脱离透析[7]。此外，病理类型对肾功能恢复也有影响，但由于本中心肾活检比例低，未将病理类型纳入研究。有报道显示轻链管型肾病患者的预后较其他病理类型患者更差[2]。

本研究仍存在一些不足。首先，本研究为单中心回顾性研究，可能在患者纳入过程中存在偏倚，且存在治疗方案不完全一致、随访数据缺失等问题，仍需在未来行更大规模的前瞻性队列研究验证本研究的结果。第二，尽管本研究纳入病例时间跨度大，反映了随时间推移MM的治疗变化、肾功能改善情况等，但部分病例临床信息缺失或不统一，如肾活检病理结果、IgH易位的伙伴基因、24 h尿M蛋白、游离轻链等，导致可统计病例数少于总队列，研究者将会继续积累有完整资料的病例以进行深入分析。

本研究探究了MM患者肾功能疗效的影响因素，发现1q21+、高钙血症及血液学疗效达到PR及以上患者是肾功能改善的独立预测指标。基线CKD分期3～5期的MM患者生存更差，治疗后肾功能改善可以弥补这一不利因素。由于单中心回顾性数据受到治疗方案不统一、随访不规律等因素影响，仍需更大规模队列研究验证这一结果。随着治疗方案不断更新、ASCT率提高，肾脏疗效对疾病预后的意义需要持续关注。
